# The Interstitial Pathways as the Substrate of Consciousness: A New Synthesis

**DOI:** 10.3390/e23111443

**Published:** 2021-10-31

**Authors:** Christopher W. Tyler

**Affiliations:** 1 Smith-Kettlewell Eye Research Institute, San Francisco, CA 94115, USA; cwt@ski.org; 2 Division of Optometry and Vision Sciences, School of Health Sciences, City University of London, London EC1V 0HB, UK

**Keywords:** consciousness, substrate, brainstem, thalamus, intralaminar nuclei, claustrum, subcortical, working memory networks, Integrative Information Theory (IIT), seizures

## Abstract

This paper considers three classes of analyses of the nature of consciousness: abstract theories of the functional organization of consciousness, and concrete proposals as to the neural substrate of consciousness, while providing a rationale for contesting non-neural and transcendental conceptualizations of consciousness. It indicates that abstract theories of the dynamic core of consciousness have no force unless they are grounded in the physiology of the brain, since the organization of dynamic systems, such as the Sun, could equally well qualify as conscious under such theories. In reviewing the wealth of studies of human consciousness since the mid-20th century, it concludes that many proposals for the particular neural substrate of consciousness are insufficient in various respects, but that the results can be integrated into a novel scheme that consciousness extends through a subcortical network of interlaminar structures from the brainstem to the claustrum. This interstitial structure has both the specificity and the extended connectivity to account for the array of reportable conscious experiences.

## 1. Introduction

The primary issue addressed here is the neural substrate of consciousness. Many aspects of consciousness can be distinguished, but the present account is focused on what Block [1] terms ‘phenomenal consciousness’, the vivid, self-reportable aspect of private, subjective experience. In this respect, it is well recognized that not all neural activity in the brain is accompanied by phenomenal consciousness. A critical requirement in determining the substrate is therefore to identify the aspect of neural activity that differentiates the conscious from the non-conscious form. A number of candidates have historically been considered for this boundary condition:

1. Neural spiking vs. graded activation in general;
2. Long-range transmission of information between cortical regions (via spiking activity);
3. Synchronous activation of populations of neurons in oscillatory modes;
4. Neural activity in a particular cortical region;
5. Neural activity in a particular subcortical structure;
6. Neural activity in a particular brainstem region;
7. Neural activity across many cortical regions that reaches a specific level of complexity;
8. Non-specific electrical activity at a sub-cellular level.

The new synthesis proposed here is a combination of aspects of several of these options, that the critical activity forming the substrate of consciousness is activity in the interstitial neuronal tissue connecting extended regions of cortex, subcortical, and brainstem nuclei. Thus, the proposal is that all neural spiking activity in this extended structure, which mediates long-range communication among cortical regions and with subcortical structures, is the activity that carries phenomenal consciousness, while neural activity in other brain structures does not. The largest component of this interstitial network is the subcortical monolayer neural sheet that forms the claustrum (which was previously proposed to be the neural substrate for consciousness by Crick and Koch [2], but seems to have lost favor in the present century), with another key component being the ascending reticular activating system (ARAS) of the brainstem (which was highly touted as critical for consciousness in the 1950s, stemming from the studies of Moruzzi and Magoun [3]).

The organization of this paper is therefore to consider how each of the eight options listed above are deficient as comprehensive theories for the substrate of consciousness, and then to develop the strengths of the interstitial theory. Those who are already convinced of the deficiencies of the eight previous approaches may simply skip to the latter section.

## 2. A Note on Terminology

Consciousness will be symbolized by ‘C*’, where the C denotes the neural substrate and the asterisk denotes the experiential (or phenomenological) activation of that substrate (as contrasted with the subconscious aspects of brain activity, that may play a role in the processing of conscious information but are not directly accessible experientially). As in Tyler [4,5], the present treatment avoids the term “neural correlate of consciousness” (NCC), in that many aspects of neural activity might *correlate* with phenomenal experience without being causally involved as its physical substrate. The preferred term here is the “neural substrate for conscious processing” (NSCP), meaning the neural activity that is the *sine qua non* for direct phenomenal experience. As such, the NSCP is a subset of the activity that correlates with C*, but it is defined by the necessary and sufficient properties that, without this activity, the organism has no C*, and it is the only activity that provides for C* in any form. The NSCP is thus effectively the ‘Holy Grail’ of C* studies, whereas matters such as electroencephalographic (EEG) *correlates* of C* recorded from the scalp may provide means for further investigation, but do not constitute its substrate in any sense.

## 3. A Definition of Consciousness

It does well when developing an analysis of a phenomenon to attempt a definition of the subject matter under investigation. The present treatment is focused on what Block [1] terms “phenomenal consciousness”, the direct experience of being vividly aware of the flow of events (as contrasted with “access consciousness”, which corresponds to the information content of mental operations controlling behavior, as in the Integrated Information Theory of Tononi [6]). Searle [7] provides an operational definition of phenomenal consciousness as follows: “By consciousness I simply mean those subjective states of awareness or sentience that begin when one wakes in the morning and continue throughout the period that one is awake until one falls into a dreamless sleep, into a coma, or dies or is otherwise, as they say, unconscious.” I would extend the wake/sleep distinction for the consciousness definition here to incorporate the distinction of “working memory” [8], or operational thought, in that consciousness is “what it is like” to imagine or think about sensory or memory contents at a given moment (as contrasted with all the possible things in memory that we could be thinking about but are presently out of awareness). This specification marks a major distinction between the direct operations of phenomenal thought itself, as opposed to the neural organization that is available to contribute to thought (similar to Block’s “access consciousness”). All definitions of consciousness are ultimately either ostensive or tautological, but it is hoped that these descriptions help to define the matter at hand relative to the reader’s own phenomenal experience.

Block’s concept of access consciousness itself seems ill-defined, however, because he allows that it can in principle exist without incurring phenomenal consciousness. In this sense, it would constitute a form of unconscious information processing that is not distinguishable from what could occur in a (biological or computational) neural network. Indeed, Block’s access consciousness thus reads as very close to the functional concept of “working memory”, the set of conceptual processes that control speech and behavior. As such, they can be investigated empirically without reference to the conscious experience of the individual under investigation. The present treatment, on the other hand, is focused on the basic set of phenomenally experienced properties of consciousness (which are necessarily those of the author as a consequence of the privacy restriction, but laid out in a form that, it is hoped, will resonate with the experience of the reader).

## 4. Types of Analyses of Consciousness

Analyses of consciousness (C*) seem to fall into three classes: abstract theories of the functional organization of C*, concrete proposals as to the neural substrate of C*, and non-neural proposals of the transcendental nature of C*, many relating it to the formalisms of quantum physics. The quantum physical approaches (such as [9,10,11,12], etc.) were analyzed by Tyler [4,5] and were shown to be based on a fundamental misconception of the role of C* in quantum physics, in that the physics is a product of the conceptualizations of human C*, rather than C* being an elaboration of non-local quantum processes. In particular, the physicists treat the wave function that underlies all subatomic entities as an objectively specified probabilistic function (though not directly observable, except by interactions that destroy it). The misconception is that probability is an inherently mentalistic construct based on the past history of interaction events, and has no physical counterpart outside the mind of the physicists (or their surrogates, the computers they have programmed). Thus, this analysis shows that the only role for C* is in designing and interpreting the physical experiment, rather than being connected to the quantum realm [4,5]. Consequently, these misleading quantal approaches will not be treated further here.

Another flavor of the non-neural substrate of C* is the class of field theories (e.g., [13,14,15,16,17,18]), which come in two flavors of substrate for C*: quantal fields and electrical fields. For either kind, there is not sufficient resolution to account for the 1 gigabit information capacity of our (unitary) visual experience (within the 1 kHz temporal resolution constraint of the neural processing to which it is supposed to be coupled). This is calculated from the 1 millisecond information rate of signals from the 1 million optic nerve fibers carrying signals from the eyes to the brain, without even considering the corresponding information rate of the auditory and tactile system. The complexity of a 100 billion neuron system is more than sufficient to account for this information processing and integration capacity. In view of space limitations, these hypotheses are also given no further consideration.

## 5. Functional Organization Theories

Theories of consciousness that focus on its functional organization are generally little concerned with its neural substrate. Examples include the Global Workspace theory [19], the Neuronal Global Workspace theory [20], the Network Theoretic Global Workspace theory [21], and the Integrated Information Theory (IIT) [6]. A primary issue to be addressed by each of these theories is that some core form of neural activity is associated with phenomenal C*, while other forms of neural activity, indistinguishable in terms of local neural spiking or intracellular voltage activity, proceed during periods of non-C*, or in the parts of the brain that are processing aspects of the current situation not reportable as involved in the current phenomenology of C*. Probably the most elaborated of these proposals in terms of analysis of the neural substrate is the last-named, IIT, so that will be considered in detail.

**Figure 1:**
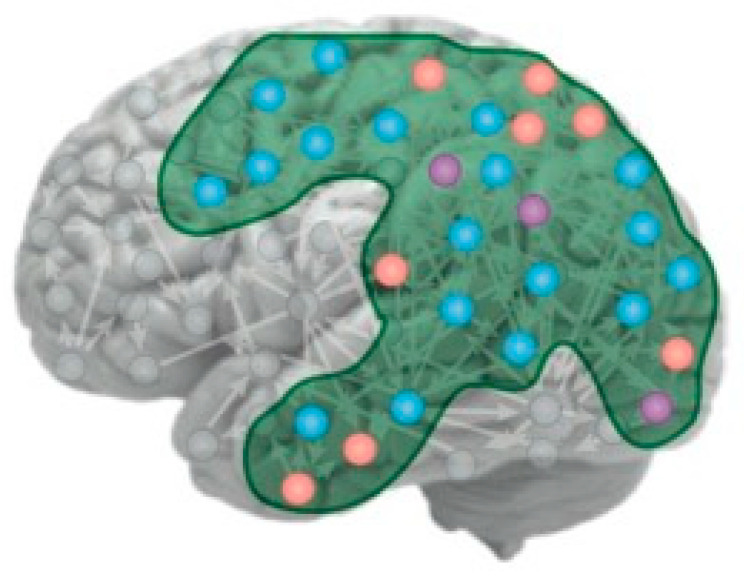
Depiction of the IIT concept of the substrate of C* as a cortical manifold. The variously colored nodes represent different forms of neural activity: orange: high firing, blue: low firing, purple: bursting, all of which could hypothetically participate in the substrate of C* (from Tononi et al., 2016).

The focus of IIT is that C* is a property associated with a particular set of neural relationships that they term its “intrinsic cause-effect power”, although it is not clear what distinguishes these particular forms of cause and effect from those of all the rest of the neurons in the brain (see Figure 1 for the kind of network envisaged). As Tononi et al. (2016) put it:

*“The axioms of IIT state that every experience exists intrinsically and is structured, specific, unitary and definite. IIT then postulates that, for each essential property of experience, there must be a corresponding causal property of the PSC [physical substrate of consciousness]. The postulates of IIT state that the physical substrate of consciousness must have intrinsic cause–effect power; its parts must also have cause–effect power within the PSC and they must specify a cause–effect structure that is specific, unitary and definite.…A cause–effect repertoire specifies how a mechanism in its current state affects the probability distribution of past and future states of the system.…By estimating the φ_max_ value of cause–effect repertoires at the level of both individual neurons and groups of neurons, an experimenter could thus assess at which grain size the network has most cause–effect power from its own intrinsic perspective—that is, at which level it makes the most difference to itself. IIT predicts that the elements of the PSC are to be found at exactly that level and not at any finer or coarser grain, a prediction that is empirically testable: does the firing of a single neuron make a difference to the content of experience, or only the average activity of a cortical mini-column?”*

(Tononi et al., 2016).

**Figure 2:**
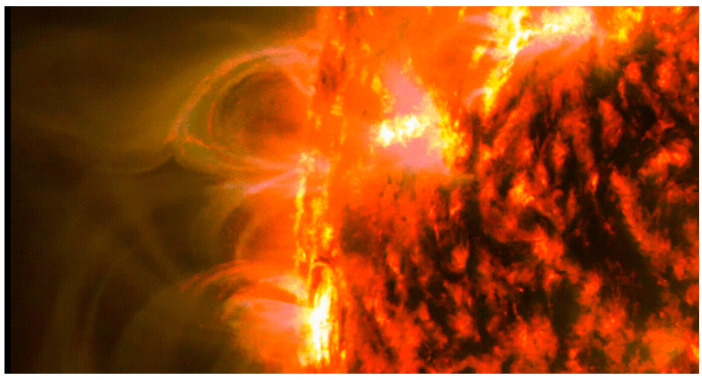
The magnetic structure of the Sun shown in two wavelengths of extreme ultraviolet light. The steamers are coronal rain along the lines of force of highly dynamic magnetic loops. Credits: NASA’s Solar Dynamics Observatory/Emily Mason. (For close-up view of solar flares on the orange surface of the Sun, see the dynamic gif from which this still image was drawn at https://www.nasa.gov/sites/default/files/thumbnails/image/coronal_rain_event_3.gif (accessed on 4 April 2021)).

Before considering the neural implications of the IIT conceptualization, we should consider the implications of its purely physical specification of C* as having an intrinsic cause-effect structure. The key aspect of IIT is that it begs the question of subjective awareness being defined only on the criterion of informational complexity. Thus, based on the IIT definition, the Sun is just as likely to house a form of C* as is someone else’s brain, and on a very similar time scale, despite the tremendous differences in size. The Sun’s rotation takes about a month, similar to the human hormonal cycles, and solar flares shoot out in local patterns of metastable “cause-effect” loops over seconds, to hours, similar to the time scale of human mental activities, with indefinitely rapid local components (see Figure 2). Thus, not only the time scale but even the spatial coherence of solar phenomena has a fractal character, going all the way from global organization at its whole spherical scale of more than a million kilometers, to local fluctuations at the finest resolution at which we are able to observe it.

In all these respects, the dynamics of solar activity seem to qualify for the IIT criteria for C* just as well as the human brain does. In fact, Tononi and Koch [22] recognize this as a limitation of the theory, and specify that it is based on the presumption of phenomenal C*, without providing any form of specification for this phenomenal basis (otherwise known as “the Hard Problem” [23]). The present analysis of the properties of the Sun is intended to highlight the limitation of this assumption, which is untestable for any system other than our own brain. In other words, they do not propose a solution for the Hard Problem, but circumvent the issue by making the phenomenology a prior assumption of their conceptualization. As expressed in the axiomatic system of Tononi et al. [24]: “IIT … starts from the essential phenomenal properties of experience, or axioms, and infers postulates about the characteristics that are required of its physical substrate.” Thus, although the “information” of IIT is analyzed as traditional mathematical information [6,22,24,25,26], it is defined as phenomenological information in an unwarranted philosophic equivalence that appears to legitimate their analysis of brain properties. The majority of the exposition is focused on the information structure of the candidates for the “physiological substrate for consciousness (PSC)”, such as its “intrinsic cause–effect power” and “maximally irreducible cause-effect structure.” [24]. (Curiously, they treat the specification “maximally irreducible” as synonymous with “maximal”, whereas something that is irreducible is by definition the minimal degree of that entity).

However, to be consistent with their definition, the analysis should focus on the information structure of the phenomenology of C* rather than of the physiology of the brain. Of the information structure of C*, IIT says only that: (1) “The axiom of information states that experience is specific, being composed of a particular set of phenomenal distinctions (*qualia*), which make it what it is and different from other experiences”; and (2) “The axiom of composition states that experience is structured, being composed of several phenomenal distinctions that exist within it.” Aside from being largely redundant with each other (see [5]), these postulates imply very minimal constraints on the structure of the phenomenology of experience (see [27]). It seems unarguable that phenomenal experience consists of a set of experiential distinctions known as *qualia* (except to the degree that they form a continuum of experiential qualities), but it is hard to see how *qualia* have any kind of intrinsic cause-effect structure, or power, or in what sense *qualia* are irreducible. *Qualia* simply exist; they do not cause any effects, and they are not caused by any other *qualia*.

The other property of C* that is not captured by the intrinsic cause-effect structure of IIT is its referentiality, or what is often termed “intentionality”, which is its property of representing something. We are not simply conscious, but always conscious of something. This is why the searchlight analogy for C* is so compelling, because a searchlight is not simply a light, but a beam that has a source and a target that is being illuminated. It therefore embodies the requisite structure of referentiality in a way that is not captured either by the local distributed cause-effect structure of IIT or by what we know of the corresponding structure of solar dynamics. Moreover, unlike the brain, the Sun has no known inputs (other than the occasional comet). Thus, it would have no vocabulary of ‘experience’ on which to draw for its source of references. Even if the Sun were conscious in principle, it would have nothing to be conscious of, so would not be conscious in the sense we are familiar with.

The criterion value for integrated information to become conscious, which is denoted Φ in IIT [24], is defined as “the minimum distance between an intact and a partitioned cause–effect structure”, by which the authors mean a structure that conforms to the postulate that C* is a unitary experience, as contrasted with the multiple sub-networks into which that structure could be partitioned, which would therefore not meet the unitary criterion. From these quotes it can be seen that Tononi’s IIT concept of the substrate of C* is a tautological form of Identity Theory. The conceptual criterion for whether a network of neurons qualifies as this substrate is whether it conforms to the property of having a unitary structure, but the unitary structure is what defines a network in the first place.

Insistence on this simplistic form of “cause-and-effect” identity evades the dual-aspect analysis of [28] and [29], in which the Hard Problem of C* is addressed by the recognition that the internal and external viewpoints of the same process may have very different describable properties, despite their common origin. A core example is the continuity of perceived visual space, such as the clear blue sky, despite the discrete receptor array conveying the information to the brain, or of an auditory tone, despite the discrete neural spikes into which the sound is encoded. In particular, the S-cones that support the perception of blue are about one tenth of the density of the other cone types, which is the reason for choosing the blue sky as an example. On this basis, therefore, uniform blue fields should be seen as an array of isolated blue spots in a yellowish field derived from the stimulation of the intervening higher-density array of M- and L-cones, rather than the uniform blue field of our experience. (In this respect, my own introspection does not support the analysis of [26], that the experience of visual space consists of an array of spots of various sizes. Instead, the space of the visual field or of the surface of an object is perceived as a continuum with overlaid patches of color, which bears no formal relationship to the cause-effect grid network of interacting units that they develop as its proposed substrate.)

Thus, the perceptual properties of the experiencer may deviate from the physical properties observed by the scientist recording from the brain. This viewpoint difference does not vitiate the underlying identity, just as the view of the road from a driver in a vehicle differs in its nature from view of the traffic helicopter of the identical scene, but it does dispute the identity of properties in theories such as IIT.

Moreover, the IIT criterion for whether each neuron or local neural circuit in the brain belongs to the network involved in C* is to test each one to determine if it makes a difference to the phenomenology of C*. The concept of performing such a test on every one of the 100 billion neurons in the human brain is, of course, laughable, not only because it would require unethical brain surgery, but because it is neurophysiologically impossible to reach every neuron, which would take many lifetimes even with the most advanced foreseeable technology, because the “unitary structure” of C* changes from moment to moment, and moreover because the change attributable to any one neuron is unlikely to be phenomenologically detectable for all but the most extreme cases.

## 6. Identifying the Neural Networks Involved in C*

**Figure 3:**
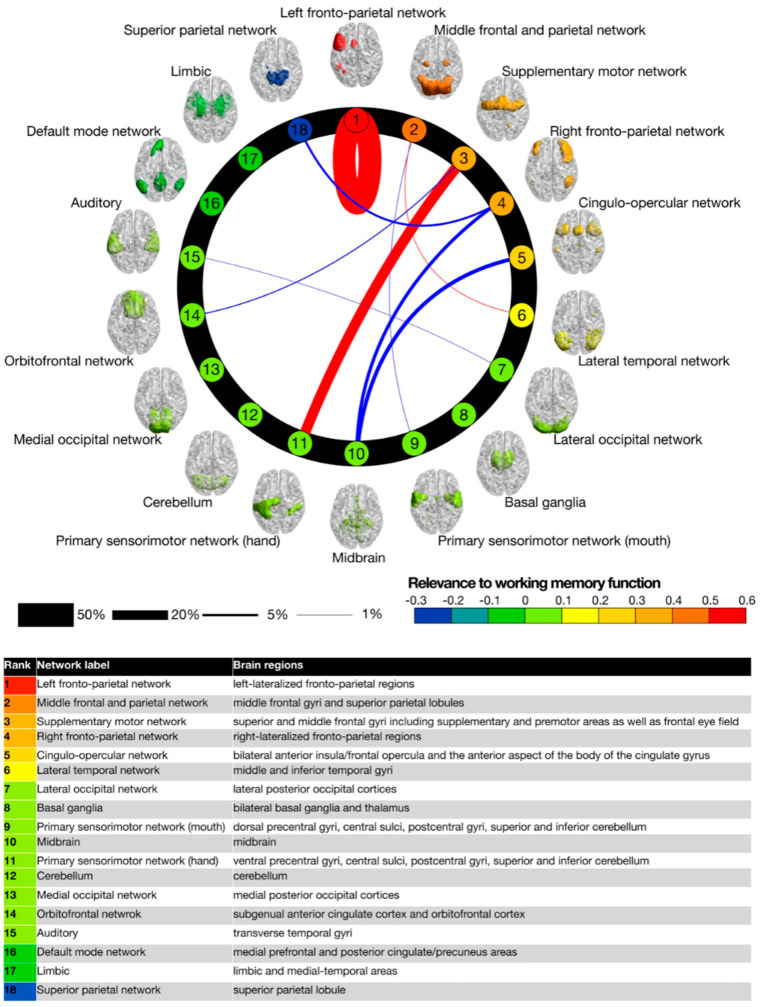
The strength of intrinsic functional connectivities among 18 brain networks during a working memory task. The cortical networks defined by whole-brain fMRI (colored brain regions) are all distinct and functionally independent at this level of resolution [31]. Each one could qualify as the substrate for C* by the IIT criterion (since each undoubtedly has component sub-networks). However, the connections for the working-memory task whose connectivity is shown here, which is the task most closely identified with phenomenal C*, is primarily restricted to the unilateral left fronto-parietal network, together with weak connectivity in motor control networks (from [30]).

Thus, the concept of identifying the PSC (or NSCP) by the neural perturbation approach outlined by Tononi et al. (2016) is both theoretically and practically barren, even if the IIT concept of C* itself were not tautological. However, even the concept of what distinguishes conscious from non-conscious neuronal networks is self-referential and moot, since this distinction is only defined in relation to the phenomenological report of the unitary property of C* (as contrasted with its multiple subnetworks). This criterion is inherently inadequate because each of those subnetworks is, *ipso facto*, itself a unitary network that could be identified as the PSC. Moreover, there are many identifiable brain networks (e.g., those identified in Figure 3) that are candidates to qualify for such empirical assessment. The Yamashita, Kawata and Imamizu study [30] is a prime example of how human brain networks can be identified by functional connectivity analysis in specific behavioral tasks, which is determined by specialized processing of functional Magnetic Resonance Imaging (fMRI) activity. Each depicted network in Figure 3 is thus defined objectively without reference to any phenomenology. Although it can only be so defined at the millimeter resolution of fMRI rather than the micrometer resolution of the posited neuronal-level networks, fMRI network analysis is an excellent place to begin the identification of the neural substrate of human C*, and indeed matches the conceptual resolution of the illustrations such as Figure 1 [22].

In the neural connectivity study of Yamashita et al. [30] they assessed the intrinsic connectivity among 18 previously identified brain networks during learning of a three-back working-memory task. The performance improvement from this learning was almost entirely attributable to the self-interaction of the dorsolateral prefrontal network (out of 324 possible excitatory connections among the 18 networks, when self-connections are included), with a few other weak contributions such as between primary and secondary motor cortex (see Figure 3), and some weak inhibitory connections. This result gives interesting insight into the functionality of working memory as a substrate for C*, with the primary effects localized to a cortical region that has been strongly associated with working memory in past studies, the *left* frontoparietal nexus (with a remarkable dissociation from its right-hemisphere counterpart). Beyond its stated aims, this study therefore provides a meaningful approach to identifying the vaunted cause-effect structure of human C*, and one that is explicitly based on the phenomenal nature of C*, rather than treating it as axiomatic.

Note, however, that this study [30] does not distinguish the specific role of the left-frontoparietal network in the functions of C*. Moreover, the focus on directed connectivity through fMRI also has the limitation that it only identifies networks at the ~1 s temporal delay available to fMRI assessment. Much of neural causality operates at delays shorter than this, so they would be missed by this analysis and only appear as instantaneous correlations. Thus, other brain regions may be involved in the working memory task at shorter delays than the one identified in Figure 3.

What is unavoidable is the psychophysiological parallelism of identifying the particular network that underlies phenomenal C*, since the phenomenology is its sole identifying characteristic. However, rather than attempting to proceed by the IIT strategy of miniscule (~10^−10^!) perturbations of the phenomenology in relation to the switching effects of single neurons, the large-scale networks involved can be identified by the complete switch of C* between on and off, or similar gross manipulations of the phenomenological state. Such switches include the short-term loss of C* in petit-mal seizures, the switch from daily C* to unconscious deep sleep, the switch from unconscious deep sleep to the dreaming state, the switch from daily C* to anaesthetically induced un-C*, and so on.

## 7. Exclusion of Cortical Regions as the Substrate for C*

It is important to make the distinction here between brain regions that are the true substrate of C* *per se* and those that are providing the replaceable *contents* of C*, and hence may be differentially activated during periods of continuous C*. Thus, the primary datum defining C* is that most people are awake all day and asleep at night, so to this extent the brain region that is the substrate for C* *per se* should be one that remains active all day and switches off at night. The main brain region whose activity matches this activity level is the thalamus [32] and its projections over the thalamocortical radiations through the internal capsule, external capsule and related structures [33], which a fortiori should be associated with the substrate for C* *per se* (as previously inferred by [34] and [35]). (A more nuanced analysis of the inferences from sleep is provided in Section 10).

The most apt analogy may be with the searchlight which, though often invoked in regard to cortical activation, is rarely elaborated in terms of its metaphorical structure. Any physical searchlight consists of three components: (a) the steerable light source, (b) the energy-transmissive light beam, and (c) the target region. It is suggested that these three components are meaningful aspects of the neural substrate of conscious perception (NSCP). At the corresponding levels, there are (a) the selective role of attention whereby the current contents of awareness, of working memory, of the “global workspace”, etc., are designated on the basis of the preceding components of the stream of activation, (b) the transmission of the selected contents to the local region(s) of cortex where they are housed, or represented, and (c) the activation of the relevant neurons in that cortical region above their baseline (non-conscious) level. To this we should add, although it stretches the analogy somewhat, the return of the cortical information to a central source that forms the actual substrate for the perceptual experience, the true NSCP. This metaphor constitutes a structured hypothesis of the mechanism of C* and, as such, evokes the reverberation hypothesis of the mid-20th century neurologists [3].

Thus, although the neural activity of the attentional searchlight may be on continuously except when asleep, the brain regions providing the contents of C*, on the other hand, should be active only during the times when the specific properties coded in a given region of the cortex are currently in the foreground of C*. Thus, to first approximation, each local region of codes for a particular aspect of conscious experience, such as perceived motion in the occipito-temporal motion area, or basic auditory tones in the Heschl’s gyrus. As has been amply demonstrated by functional MRI studies, the activity in these areas covaries strongly with the conscious perception of such sensory qualities, and they are functionally silent during other conscious experiences. This line of reasoning refutes the contention by Kanwisher [36] that the NSCP is in the cortex, for the continuous persistence of C* *per se* transcends the local specification of the contents of C*. Moreover, while the local cortical activity may thus be necessary for C*, it is unlikely to be sufficient to support the continuous awareness of fleeting contents.

This issue arises particularly in cases where the substrate of C* is identified as corresponding to the cortical default-mode network (DMN), such as [37,38,39]. The contents of C* when the DMN is activated have not yet been securely identified, but it should be clear that the switching in and out of the DMN during task-relevant vs. task-irrelevant relaxation periods all occurs during waking C*, so this excludes the cortical network of the DMN as a candidate for the substrate of C*, since much of that network is strongly suppressed during the task-relevant periods of C*.

## 8. Evaluation of Concrete Proposals for the Substrate of Consciousness

**Figure 4:**
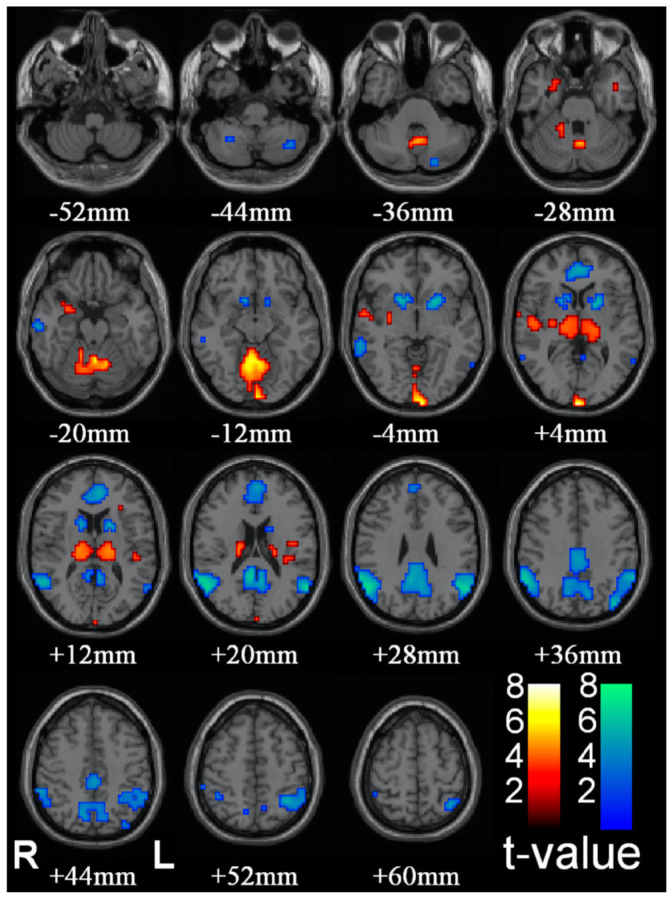
Changes in brain activity during epileptic absence seizures (from [40]).

Blumenfeld [40], see [41], develops a proposal for the substrate of C* based on clinical indices of observable disruptions in the state of C*, such as epileptic absence (petit-mal) seizures. Group data of the effect of such seizures are reproduced in Figure 4. The logic of the analysis is that absence seizures represent a reportable and behaviorally corroborated loss or steep reduction of C*, though they are not accompanied by the loss of muscle tone associated with sleep. The subject will remain in an upright seated posture, for example, but stop speaking for the few seconds which comprise the duration of the seizure, and exhibit confusion on returning to C*. Thus, brain regions showing a decrease in activation during this period should be associated with the normal C* that is disrupted during this period, while those showing increases may best be interpreted as being inhibitory centers.

The brain regions showing consistent reductions in fMRI activation during petit mal absences are the ventral striatum in the region of the nucleus accumbens and the caudate nucleus (blue patches from −4 to +12 mm in Figure 4) and medial and lateral components of the DMN (blue patches from +4 to +60 mm in Figure 4). Remarkably, some brain regions show marked increases in fMRI activation during the absences, notably the thalamus (orange patches from +4 to +12 mm in Figure 4) and what appear to be some medial structures in the cerebellum (orange patches from −20 to −12 mm in Figure 4). These are probably best interpreted as inhibitory brain loci, with the thalamic inhibition emanating from the reticular nucleus of the thalamus, which is an inhibitory net surrounding the thalamus that acts as a gating mechanism to control sensory input to and from the cortex.

Components of both the dorsal and ventral striatum have been associated specifically with the conscious/non-conscious boundary in perceptual tasks [42,43]. However, these forms of differential activation may not relate directly to C* *per se*, since the manipulation determines when a particular stimulus reaches the level of C*, which is then processed for the Yes response, as opposed to the No response when it is not seen. These responses require both differential motor programming and a sense of reward when seen, which are the well-established functions of the dorsal and ventral striata, respectively. However, neither involves any change on the overall conscious state of the participants, but only its contents. This kind of paradigm therefore does not address the core issue of C* *per se*.

**Figure 5:**
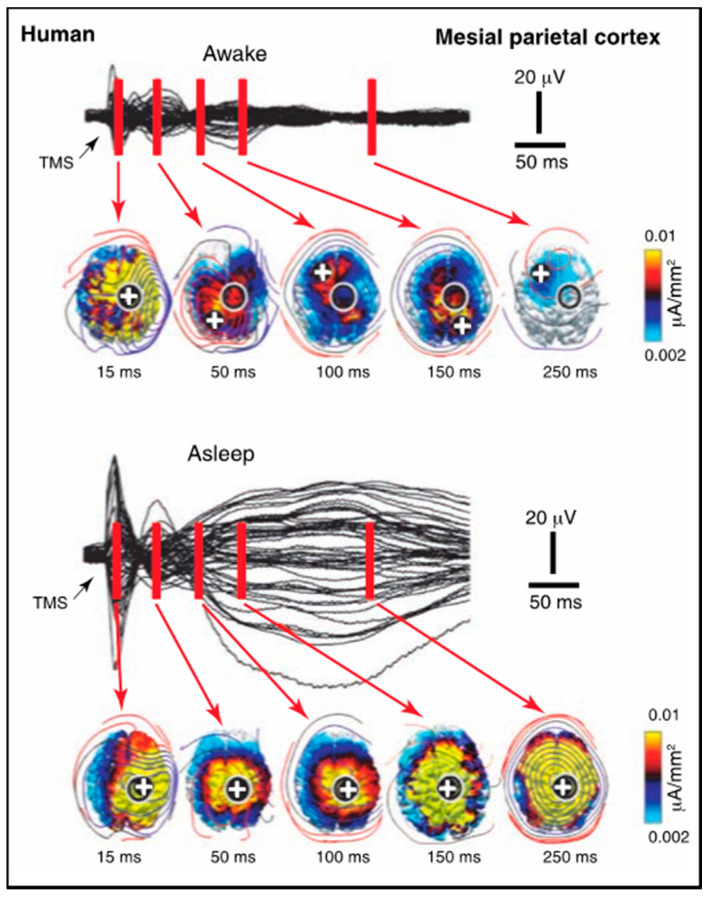
Effect of parietal-region TMS in differing levels of C*. When awake, when the response is low amplitude with a succession of local activation sites, vs. during deep sleep, when the response is high amplitude and spread across a broad region of cortex. (Figure 3C from [32]).

A paper on anaesthesia [32] also reports paradoxical effects of transcranial magnetic stimulation (TMS) on cortical activation during NREM (deep) sleep versus wakefulness. In the premotor cortex, the stimulation evokes a small local response, followed by a sequential series of activations in various other cortical regions. During deep sleep, the same stimulation evokes a large response that remains relatively local to the stimulated region. Thus, although the amplitude of activation does not correlate, or rather correlates inversely, with the loss of C* (consistent with the refutation of the local cortical activation hypothesis for C*), the absence of the spread of activation implies a loss of integrative function. On the other hand, repeating the same experiment with TMS over the medial parietal cortex (Figure 5), again produces a similar pattern of low-level sequential activation sites when awake, while producing a large amplitude signal during deep sleep that spreads over much of the dorsal cortex.

These results should be compared with the IIT criteria for C*, that its substrate should be unitary, for example. The EEG response is clearly much more unitary in the deep sleep response than the diverse cortical loci involved in the awake response. While there may be ways to avoid this implication, it clearly poses profound problems for the unitary assumption of the IIT interpretation. Equally, the much larger cortical response during the unconscious state of deep sleep again disposes of any theory in which the cortex is invoked as a component of the NSCP.

The primary characteristic of the IIT conception of C* of being unitary, or integrated, both at the phenomenological and neural level, is what is proposed as giving it the cause-effect power to form the basis of phenomenal C*. However, this identification is problematic in that the intrinsic cause-effect power of the phenomenology, which the proponents do not discuss in detail, is of a very different character than that of the neural substrate. For example, Tyler [28] proposes that C* has a superordinate form of causal relations that is emergent from physiological connectivity and that these superordinate dynamics are identical at both the physiological and the phenomenal levels, whereas the causal processes at the *local* neural level are not expressed phenomenally. However, IIT puts the emphasis on the integration of both the local and global causal relations, implying that the conscious phenomenality should match all aspects of the local neural dynamics (which does not seem to be the case, since conscious experience does not consist of trains of millisecond spikes).

## 9. An Interstitial Network for Consciousness: A New Hypothesis

In surveying the literature on the NSCP, two common themes emerge. One is that changes in C* typically involve large regions of cortex. The other is that they often involve a variety of poorly-understood subcortical structures, from the lowly brainstem up to the highest levels of subcortical strata, the claustrum. To integrate these various findings (to be detailed below), we may make use of the longstanding distinction between the experiential salience versus the phenomenal contents of C*. The key to this distinction is that the salience is univariate whereas the contents are highly multivariate. The salience is expressed in terms of the intensive dimension of variations in vividness or aliveness that we feel, in contrast to drowsiness, ending with the un-C* of sleep. The contents, of course, include the rich variety of aspects of the environment, together with the range of internally generated thoughts and states of emotions that are well established to show selective expression across that array of cortical networks, such as those identified in Figure 3. This line of reasoning makes it evident that, if the intensive/contents distinction does correspond with the cortical/subcortical division, it must be the cortex that encompasses the contents of C* and the subcortical structures to the intensive dimension of C*.

The most apt analogy may be with the searchlight which, though often invoked in regard to cortical activation, is rarely elaborated in terms of its metaphorical structure. Any physical searchlight consists of three components: the steerable light source, the energy-transmissive light beam, and the target region. It is suggested that these three components are meaningful aspects of the neural substrate of conscious perception (NSCP). At the functional level, there is (a) the selective function of C* whereby the current contents of awareness, of working memory, and of the “global workspace”, etc., is designated on the basis of the preceding components of the stream of C*, (b) the transmission of that selection to the local region(s) of cortex where the contents are housed, or represented, and (c) the activation of the relevant neurons in that cortical region above their baseline (non-conscious) level. To this we should add, although it stretches the analogy somewhat, the return of the cortical information to a central source that forms the actual substrate for the perceptual experience, i.e., the true NSCP. This metaphor constitutes a structured hypothesis of the mechanism of C* and, as such, evokes the reverberation hypothesis of the mid-20th century neurologists.

**Figure 6:**
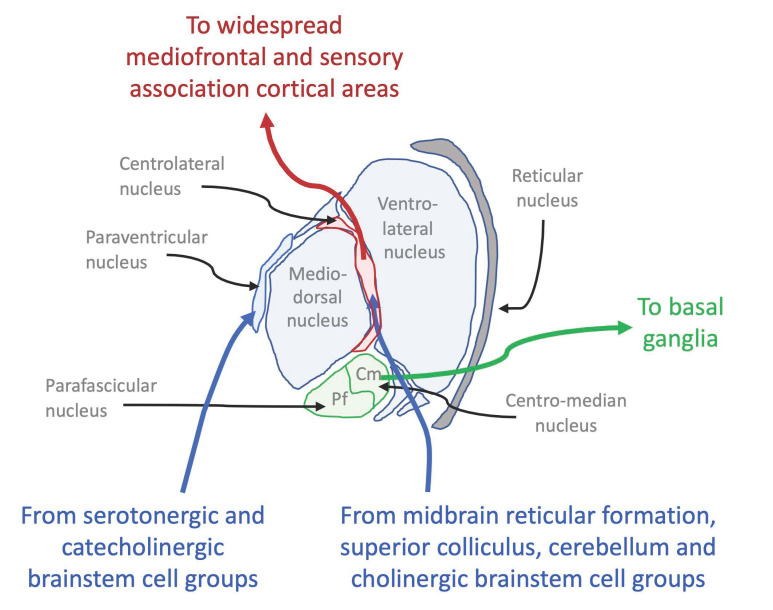
Inputs and outputs of the midline and intralaminar nuclei of the thalamus. After [49].

What, then, are the subcortical structures that have been associated with C*? Particularly in studies of anaesthesia, the strongest association has been with a network of intralaminar and reticular structures of the claustrum [44,45], the so-called “non-specific” midline and intralaminar nuclei of the thalamus, comprising the paraventricular, parataenial, intermediodorsal, reuniens and rhomboid thalamic nuclei [46,47], together with the nonspecific zona incerta lying just below the thalamus and several brainstem structures, including the pretectal area, the nucleus reticularis tegmenti pontis, and the basis pontis [38,48], all of which are suppressed in anaesthesia. These midline and intralaminar nuclei of the thalamus are delineated in Figure 6.

A prime example of the empirical use of the extinguishability criterion for C* is a paper by Koubeissi et al. [50], which found that electrical stimulation of the (left) claustrum above a certain threshold reversibly extinguished the participant’s C* for the time period of the stimulation, whereas corresponding stimulation of nearby brain regions had no such effect. This result supports the suggestions that the claustrum plays an important role in the modulation of C* *per se*, since it seems to be one of the few papers since Moruzzi and Magoun [3] to identify a brain structure that is causally involved in switching between the C* and non-C* states. That was the legendary study that identified the role of the ascending reticular activating system (ARAS) of the brainstem pons and the midbrain in controlling the sleep/wake states in cats. The fact that the claustrum, lying just below the cortex, plays a similar switching role helps to tie it in with the ARAS, through the intralaminar thalamic and corona radiata, as an integrated subcortical substrate for C*. It should be noted, however, that the suppression of C* by the activation of the claustrum appears to imply that it plays a selective inhibitory role corresponding to that of the intralaminar nucleus of the thalamus (but at a higher level of processing), rather than being the full substrate of C* alone, as originally proposed by Crick and Koch [44].

**Figure 7:**
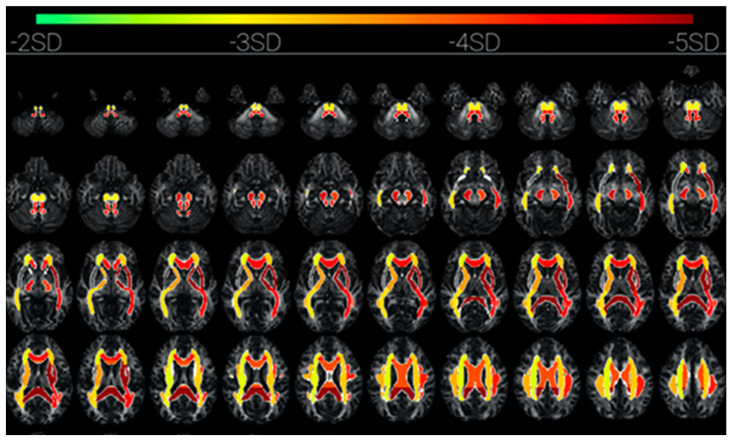
The subcortical network of reduced fractional anisotropy in a patient with prolonged loss of C* (from [51]). Strongest activation is dark red. Upper two rows: brainstem structures; middle row: predominantly the internal and external capsules, plus the splenium; lowest row: predominantly the corpus callosum.

Some of these other structures that are revealed by diffusion tensor imaging (DTI) in cases of prolonged loss of C* due to COVID-19 encephalitis [51] are the subcortical pathways of the corona radiata, superior longitudinal fasciculus, anterior and posterior limb of internal capsule, and external capsule. These are interlaminar subcortical structures linking the intralaminar thalamic nuclei and ventral striatum with the limbic structures of the basal ganglia and the claustrum [52]. These structures are visualized in Figure 7 by the change in fractional anisotropy of the DTI during the loss-of-consciousness episode (Figure 2 from [51]). This mapping may be taken as an overview of the subcortical network involved in C* (although not all components would necessarily be involved, since shutting down the core C* would also deactivate dependent components of the complex). In the searchlight metaphor, switching off the source of the searchlight would also switch off the light beam and the illumination of the target object.

Conversely, one component of this subcortical network, the intralaminar region of the thalamus, has recently been shown to have a specific alerting function by direct stimulation while asleep [53]. Stimulation of the central lateral portion of the intralaminar region of the thalamus of anaesthetized macaques consistently awoke them from the anaesthetized state, thus extending the results of Moruzzi and Magoun [3] to this interstitial brain structure directly connected with the midbrain ARAS lying just below it.

## 10. Conclusions

This paper considers three classes of analyses of the nature of consciousness (C*): abstract theories of the functional organization of C*, concrete proposals as to the neural substrate of C*, and non-neural proposals of the transcendental nature of C*, many relating it to the formalisms of quantum physics. It shows that the quantum formalisms of the nature of C* are inherently flawed in the conception of probability as a physical entity, and thus cannot serve as a theoretical basis for human C*. It shows that abstract theories of the dynamic core of C* have no force unless they are grounded in the physiology of the brain, since the organization of complex dynamic systems, such as the Sun, could equally well qualify as conscious under such theories. This analogy highlights the fact that C* as we experience it has referential content derived from inputs to the system, which is an essential aspect missing both from IIT theory and from the Sun as a model complex system. Various other aspects of the IIT theory are also subjected to critical review. An alternative approach in which specific network connectivities are assessed in a C*-based working memory task reveals the part of the neural substrate of C* accessed in this particular case, although this analysis is still incomplete due to the limitations of fMRI causal connectivity.

An overview of the wealth of studies of human C* since the mid-20th century concludes that many proposals for the particular neural substrate of C* are insufficient in various respects. However, the results can be integrated into a novel scheme that C* extends through a subcortical network of interlaminar structures from the brainstem to the claustrum. This interstitial structure has both the specificity and the extended connectivity required to account for the unified yet multifold and diverse array of experiences of reportable C*.

## Data Availability

Not Applicable.
